# Tubercle Bacilli in Blood

**Published:** 1912-11

**Authors:** 


Tubercle Bacilli in Blood.—R. Hildermann and I. Losses, *Deutsche Med. Woch.* No. 19, 1912, have found tubercle bacilli in the blood in about a quarter of pulmonary cases examined, the finding being unfavorable but not indicative of generalized miliary tuberculosis.

